# Left Atrium Volume Measured with Multislice Computed Tomography as a Prognostic Predictor for Atrial Fibrillation Catheter Ablation Outcomes

**DOI:** 10.3390/jcm13071859

**Published:** 2024-03-23

**Authors:** Jae-Hong Park, Dong-Hyun Yang, Ji-Hyun Kim, Yoo-Ri Kim

**Affiliations:** 1 Division of Cardiology, Department of Internal Medicine, Kangnam General Hospital, Yongin 17064, Republic of Korea; hh9476@naver.com; 2 Department of Radiology, Asan Medical Center, Seoul 05505, Republic of Korea; donghyun.yang@gmail.com; 3 Division of Cardiology, Department of Internal Medicine, Dongguk University Ilsan Hospital, Goyang 10326, Republic of Korea; kimjihyun@dumc.or.kr; 4 Division of Cardiology, Department of Internal Medicine, College of Medicine, Chonnam National University, Gwangju 61469, Republic of Korea

**Keywords:** atrial fibrillation, left atrial volume, multislice computed tomography, recurrence

## Abstract

**Background:** Current guidelines consider atrial fibrillation (AF) type as the prognostic factor for a recommendation of catheter ablation. We aimed to determine whether LA and LA appendage (LAA) volumes measured using multislice computed tomography (MSCT) were related to long-term outcomes in AF following radiofrequency catheter ablation (RFCA). **Methods:** We evaluated 152 consecutive patients with drug-refractory AF (median age, 55.8 ± 9.6 years), including 110 male patients, who underwent RFCA in a single center. All patients underwent MSCT imaging for anatomical assessment. The endpoint of this study was documented AF recurrence after RFCA. **Results:** The overall procedure success rate was 77.6% (*n* = 118) during a mean follow-up period of 12.6 months. The LA volume was significantly larger for those who experienced AF recurrence after RFCA than for the patients without recurrent AF after the procedure (153.8 ± 29.9 mL vs. 139.2 ± 34.1 mL, *p* = 0.025). However, LAA volumes were nearly equivalent between the patients with and without AF recurrence after RFCA (16.2 ± 6.3 mL and 14.7 ± 6.5 mL, respectively; *p* = 0.235). LA volume ≥ 153.2 mL was the optimal cutoff value for estimating AF recurrence after RFCA, with 94% sensitivity and 66% specificity. LA volume remained an independent predictor of both AF recurrence and permanent AF. **Conclusions:** LA volume as assessed by MSCT might be helpful for identifying patients likely to achieve successful AF ablation. LA volume ≥ 153.2 mL, but not LAA volume, showed good accuracy in predicting AF recurrence after RFCA.

## 1. Introduction

The prevalence of atrial fibrillation (AF), which is the most common arrhythmia in adults [1], is increasing simultaneously and in proportion to the increasing age of populations [2]. AF is mainly caused by certain triggers, abnormalities in the structure and electrical activity of the heart, overactive nervous system and hormone levels, as well as genetic factors, which lead to the spontaneous development of AF [3]. AF induces thromboembolic stroke, heart failure, and myocardial infarction, and is linked to increased mortality [4]. Pulmonary vein (PV) isolation via radiofrequency catheter ablation (RFCA) is a dedicated therapeutic alternative for rhythm control in patients with drug-refractory AF. RFCA is an acceptable and appropriate alternative to pharmacotherapy for the preventative method of AF recurrence in paroxysmal AF (PAF) or persistent AF (PeAF) patients [5,6]. The success rate of RFCA is about 50% in patients with PeAF and 70% in those with PAF [7]. Identifying predictors of long-term success with RFCA is critical for selecting patients who would profit most from this intervention.

A few studies have reported volume as an independent factor of recurrent atrial arrhythmia [8,9,10,11,12,13], although the AF type (paroxysmal or non-paroxysmal) is among the most consistent prognostic factors of long-term outcomes after RFCA [14]. However, whether the measurement of LA size can be used to predict success after RFCA remains unclear because of the different methods utilized to evaluate LA structure among the studies. For example, the anterior–posterior (AP) LA diameter is not the most exact indicator of a LA size [15]. Therefore, a measurement of the AP LA diameter may have limited applicability for selecting patients eligible for RFCA.

A previous study demonstrated that LA dilatation was asymmetric. The authors reported that LA enlargement in patients with AF was principally in the medial–lateral and superior–inferior axes, as AP dilatation was constrained by the thoracic cavity [16]. Therefore, LA volume measured using multislice computed tomography (MSCT) was a successful parameter of AF ablation, unlike the AP LA diameter on transthoracic echocardiography [8]. A three-dimensional (3D) imaging method using MSCT provides a more precise calculation of the LA size and exhibits a higher prognostic RFCA outcome [8,11,13]. Additionally, LAA volume has also been examined as a strong recurrence predictor after RFCA [17].

MSCT allows for a better description of the complicated LA anatomy and improves the accuracy of electro-anatomic mapping with 3D image-merging techniques. However, few studies evaluated both LA and LAA volumes as true predictors of AF recurrence after RFCA. Therefore, we aimed to determine whether LA and LAA volumes calculated using MSCT were related to long-term outcomes after RFCA.

## 2. Methods

### 2.1. Study Population

We retrospectively analyzed data from 152 consecutive patients who underwent RFCA for symptomatic drug-refractory AF at Asan Medical Center from 1 August 2011 to 31 August 2012. The inclusion criteria were as follows: adult patients with symptomatic AF refractory to at least one class 1 or 3 antiarrhythmic drug. We excluded patients with significant valvular disease, prior ablation, or cardiac surgery, as well as those unable to undergo an MSCT scan due to renal insufficiency or contrast allergy. Comprehensive data collected included demographics, physical examination findings, clinical history, and detailed transthoracic echocardiography metrics delivered within one month before the ablation procedure. Antiarrhythmic medication cessation occurred 48 h before RFCA to minimize their potential influence on procedural outcomes.

### 2.2. Multislice Computed Tomography

**Figure 1:**
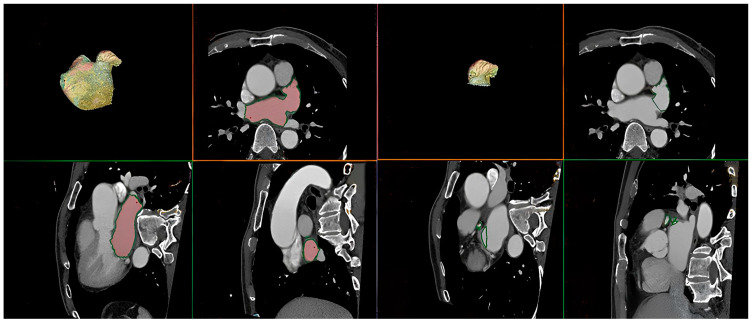
Measurement of left atrial and left atrial appendage volumes using multislice computed tomography images with a standard workstation (AW Server 2), performed by a radiologist and a cardiologist. This illustration showed how we measured left atrial and left atrial appendage volumes from computed tomography images.

All patients underwent electrocardiography (ECG)-gated 128-channel MSCT with a second-generation dual-source computed tomography system (Somatom Definition Flash; Siemens Healthcare, Erlangen, Germany) for the estimation of PV anatomy, calculation of LA and LAA volumes, identification of LA thrombi, and integration of electro-anatomical mapping. Image scanning was carried out from the aortic arch to the diaphragm in a single breath-hold through ECG synchronization gated to the cardiac cycle, and the acquisition was ECG-gated after the bolus injection of iodine contrast medium based on body weight. Images were acquired at 70–80% of the RR interval just before the atrial systole [18]. 3D images of the left atrium and the map of PV were uploaded to the AW Server 2 volume-rendering software (GE Healthcare, Waukesha, WI, USA). To determine the LA volume, LA boundaries were outlined on every axial slice, and pulmonary arteries and the LAA were excluded by drawing a continuous imaginary line. After inputting LA borders and multiplanar segmentation, outlines of LA were automatically composed and adjusted by hand while referring to a reconstructed 3D model (Figure 1). The LA volume was measured by multiplying each slice’s area and thickness and adding up the volumes of all slices. MSCT images were analyzed offline using cardiac imaging software (AW Server 2) on a standard workstation, which was performed by two experienced independent readers (a radiologist, DH Yang, and a cardiologist, YR Kim) for interobserver reliability.

### 2.3. RFCA 

The ablation procedure was performed on an individualized patient basis, adhering to the current guidelines for catheter ablation of AF. The RFCA procedure aimed to achieve durable isolation of the PVs, the primary sources of abnormal electrical triggers in AF. Three catheters were introduced, under local anesthesia and sedation, through the right femoral vein to access the left atrium via a transseptal puncture with midazolam and remifentanil sedation if needed. A multipolar diagnostic catheter was placed in the coronary sinus, and radiofrequency energy was employed for circumferential PV isolation using a 3D mapping system (CARTO^®^ 3; Biosense Webster, Irvine, CA, USA). The CARTO^®^ 3 system’s electro-anatomical maps, integrated with the MSCT-derived images, facilitated live navigation. The endpoint of PV isolation was assessed by documenting entrance and exit block. Additional ablation lines, such as cavotricuspid isthmus, LA roof, and mitral isthmus lines, were ablated based on the operator’s discretion. In PeAF, direct current cardioversion restored sinus rhythm post-RFCA when required.

### 2.4. Clinical Outcomes

The primary endpoint of the study was AF recurrence. We defined AF recurrence as follows: (1) AF documented for at least 30 s using a 12-lead ECG or 24 h Holter monitoring and (2) direct current cardioversion or repeat RFCA due to AF recurrence after the blanking period, defined as the first three months after the index ablation required for atrial tissue healing. We followed up with patients for a minimum of 12 months after RFCA. Additionally, 12-lead ECG and 24 h Holter monitoring were performed at 3, 6, and 12 months after RFCA. Furthermore, event recordings and 48 h Holter monitoring were implemented to evaluate patients with suspicious symptoms. All patients restarted taking the oral anticoagulation on the procedure day, according to the CHADS_2_-VASc score [19]. Anti-arrhythmic drugs were administered for the first three months before RFCA or discontinued immediately after the procedure at the treating physician’s discretion [20].

### 2.5. Statistical Analysis

Continuous variables are revealed as means ± standard deviation and categorical variables are revealed as percentages with frequencies. The former are compared using Student’s *t*-test for normally distributed data and the nonparametric Mann–Whitney *U* test for non-normally distributed data. The latter are compared using the two-tailed Fisher’s exact test or the chi-square test. The Cox proportional hazards regression model was used to determine variables related to AF recurrence after RFCA. Variables with a *p*-value < 0.05 as statistical significance in the univariate analyses were inserted in multivariate analyses to determine independent AF recurrence predictors, with probability values for inclusion and exclusion defined as 0.05 and 0.10, respectively. The adjusted hazard ratios (HRs) with 95% confidence intervals (CIs) were calculated. All possible confounders were included, regardless of statistical significance. We just included the following variables simultaneously into the model: age, body mass index, sex, diabetes mellitus, hypertension, AF type, LA volume, LAA volume, LA volume/LAA volume ratio, left ventricular ejection fraction (LVEF), and the number of prescribed anti-arrhythmic medications. Kaplan–Meier analysis was used to describe the recurrence of AF over time across the strata of LA and LAA volumes with the log-rank test. Furthermore, receiver operating characteristic (ROC) curve analysis was used to decide the optimal LA volume cutoff value predicting AF recurrence, which was defined with the highest sensitivity and specificity. In this study, statistical significance was considered at *p*-values less than 0.05. IBM SPSS Statistics for Windows, version 22.0 (IBM SPSS Statistics, Armonk, NY, USA) was used when statistical analyses were performed.

## 3. Results

### 3.1. Baseline Characteristics

During the study period, 152 consecutive patients, with a median age of 55.8 ± 9.years, underwent RFCA for symptomatic drug-refractory AF. Male patients comprised 72.4% (*n* = 110) of the cohort, and 65.1% of the patients (*n* = 99) were diagnosed with PAF.

Baseline patient characteristics are presented in Table 1. During a mean follow up of 12.6 months (interquartile range, 8.7–18.5 months), the overall procedure success rate was 77.6% (118 of 152 patients). Study patients were categorized into two groups from AF recurrence after RFCA. Although there were no statistical differences in age, sex, and BMI between the two groups, this table showed that PAF was more frequent in the no recurrence group and the LA (left atrial) volume was larger in the recurrence group. The univariate analysis revealed that age, sex, hypertension, diabetes mellitus, and history of stroke were not associated with AF recurrence. In contrast, PAF and higher LVEF were associated with a significantly lower rate of AF recurrence after RFCA (*p* < 0.05) although the median LVEF was within the normal range in both groups.

**Figure 2:**
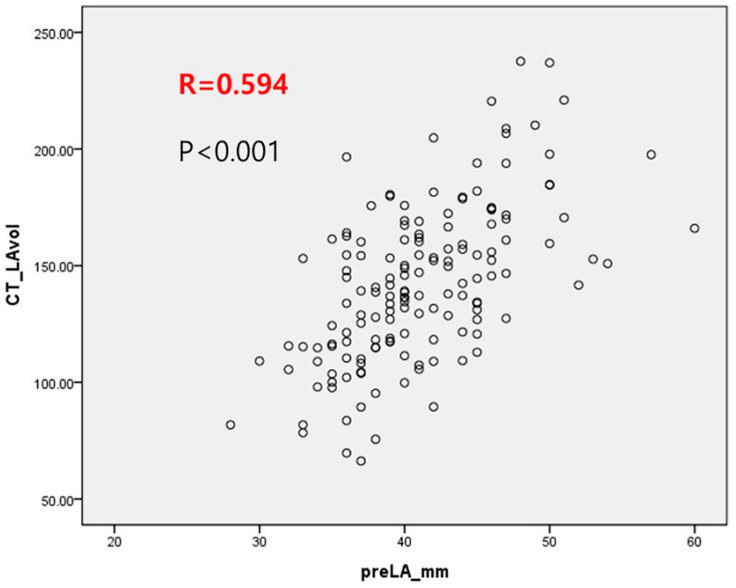
Correlation between the anterior–posterior left atrial diameter determined using transthoracic echocardiography and left atrial volume determined using multislice computed tomography.

The mean LA and LAA volumes calculated using MSCT were 142.5 ± 33.7 mL (66.3–237.6 mL) and 15.1 ± 6.5 mL (4.6–21.6 mL), respectively. The LA volume was meaningfully lower in patients with PAF than in those with PeAF (132.5 ± 30.9 vs. 161.2 ± 30.7 mL, *p* < 0.0001). Additionally, the LAA volume was lower in patients with PAF than those with PeAF (14.2 ± 5.9 vs. 16.6 ± 7.2 mL, *p* = 0.038). In this study, we found a moderate correlation between LA diameter determined using TTE and LA volume determined using MSCT (*r* = 0.594, *p* < 0.001; Figure 2).

### 3.2. Clinical Outcomes

The patients with recurrent AF after RFCA had significantly larger LA volumes than those who did not experience AF recurrence after the procedure (153.8 ± 29.9 mL vs. 139.2 ± 34.1 mL; *p* = 0.025). However, the LAA volume was comparable between patients with and without AF recurrence after RFCA (16.2 ± 6.3 mL and 14.7 ± 6.5 mL, respectively; *p* = 0.235).

**Figure 3:**
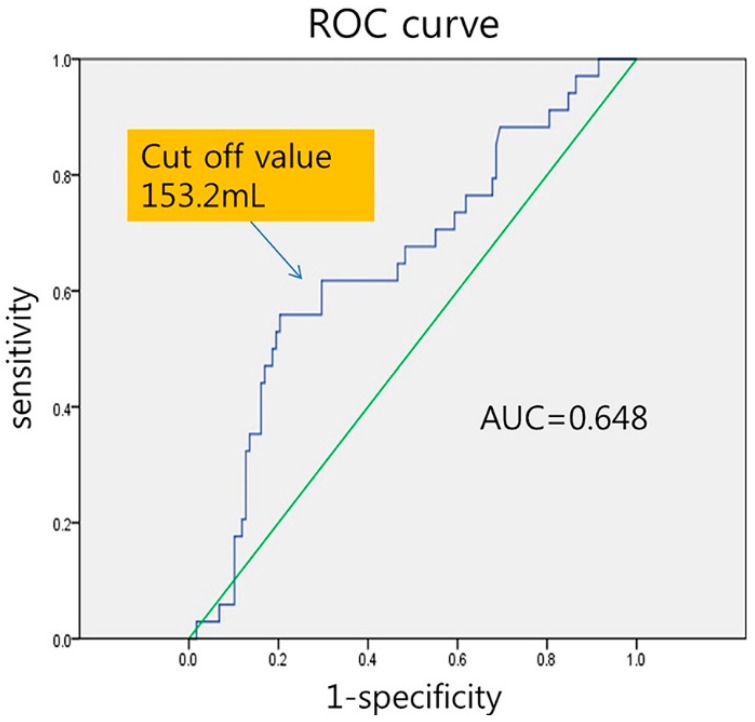
Receiver operating characteristics (ROC) curve showing the best cutoff value for left atrial volume to predict success after radiofrequency catheter ablation for atrial fibrillation.

**Figure 4:**
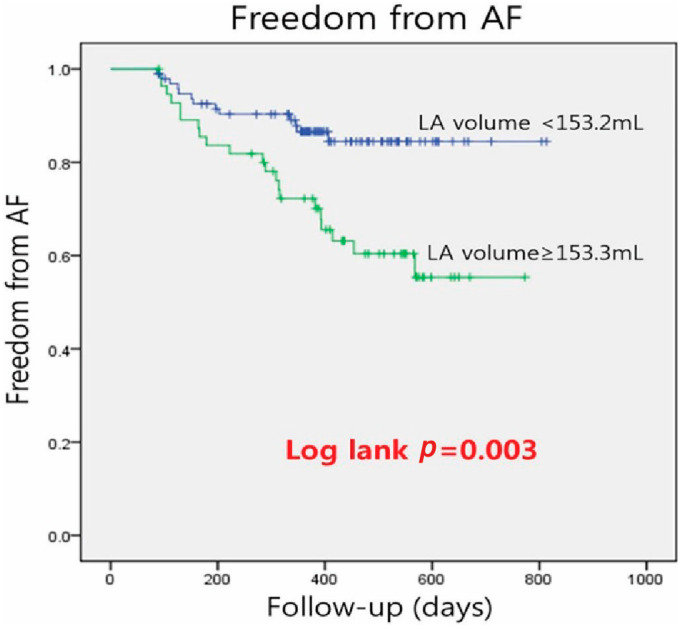
Kaplan–Meier analysis comparing a recurrence of atrial fibrillation after radiofrequency catheter ablation between patients with left atrial volume < 153.2 mL compared to those with left atrial volume ≥ 153.2 mL based on assessment with multislice computed tomography.

The ROC curve analysis showed that LA volume ≥ 153.2 mL was the best cutoff value to predict AF recurrence after RFCA, with 94% sensitivity and 66% specificity (area under the ROC curve, 0.648; Figure 3). The patients were classified into those with LA volume < 153.2 mL (*n* = 96) and those with LA volume of ≥153.2 mL (*n* = 56). Kaplan–Meier analysis demonstrated that long-term AF-free survival was greater in patients with LA volume of <153.2 mL than those with LA volume of ≥153.2 mL (84.9% vs. 61.5%, *p* = 0.017; Figure 4). The log-rank test showed a significant difference between the groups (*p* < 0.003).

### 3.3. Univariate and Multivariate Analysis for Predicting AF Recurrence after RFCA

In the final univariate Cox proportional hazards regression analysis seeking to identify factors associated with the recurrence of AF following RFCA, we ascertained that an LA volume (≥153 mL) significantly predicted recurrence, with an adjusted HR of 2.771 (95% confidence interval [CI], 1.385–5.541; *p* = 0.004), as shown in Table 2. Moreover, the presence of PeAF was associated with a higher risk of recurrence (adjusted HR, 3.944; 95% CI, 1.95–7.975; *p* = 0.001), as was a decreased LVEF (adjusted HR, 0.939; 95% CI, 0.906–0.974; *p* = 0.001). Nonetheless, the volume of the left atrial appendage (LAA) did not serve as a prognostic factor in AF recurrence post-RFCA (adjusted HR, 1.021; 95% CI, 0.976–1.068; *p* = 0.359). Subsequent multivariate analysis, which controlled for PeAF, LA volume, and LVEF, corroborated the independent predictive value of PeAF in AF recurrence following RFCA (adjusted HR, 2.759; 95% CI, 1.260–6.041; *p* = 0.011), as shown in Table 3.

## 4. Discussion

In this study, we aimed to determine whether LA and LAA volumes measured using MSCT were associated with long-term outcomes of RFCA for AF, and found that LA volume ≥ 153.2 mL was independently involved in AF recurrence after RFCA in spite of the statistical results underscoring PeAF as the most positive factor. Although there was only a positive association between PeAF and recurrence after RFCA from multivariable Cox regression analysis, we aimed to investigate AF recurrence after RFCA procedure according to LA volume in this study. The identified cut-off value for LA volume was 145 mL, and based on this value, patients were divided into two groups to assess the prognosis for each group. It was intended to explore the association between LA volume and the likelihood of AF recurrence after RFCA. However, this study did not reveal a relationship between LAA volume and long-term outcomes after AF ablation using RFCA.

The Framingham Heart Study reported that LA enlargement was an independent risk factor for newly onset AF in the general population [21]. Other studies reported that LA dilatation was a long-lasting, uninterrupted process mainly responsible for the perpetuation of AF, although LA electrical remodeling was rapidly established within hours following AF initiation [22,23]. Larger LA volumes yield larger LA surface areas and are associated with increased wall stress and thickness, which likely renders atrial tissue more resistant to the transmural lesions created during RFCA [24]. Although the RFCA treatment modality can be a curative treatment option in patients with PAF, it is associated with a reasonable recurrence rate and some potentially severe complications [25]. Proper patient selection and precise identification of LA anatomy before the procedure are crucial to improve RFCA success and prevent unnecessary procedure-related complications. A more thorough estimation of bona fide LA sizes may enhance the detection of patients with AD who can achieve long-term success after RFCA. According to the Heart Rhythm Society/European Heart Rhythm Association/European Cardiac Arrhythmia Society expert consensus statement, the collection of suitable patients for RFCA should consider age, symptom severity, AF duration, and LA diameter [26]. The AP LA diameter was shown, by Berruezo et al., to be an independent predictor of AF recurrence after RFCA [27]. In contrast, another study revealed that echocardiographic calculations of LA size did not correlate with success after AF ablation [28]. These conflicting results might be associated with interobserver variations in LA assessment. No definitive criteria were used for evaluating LA enlargement with transthoracic echocardiography (TTE). A study using both TTE and MSCT reported the utility of LA volume assessed using MSCT as a successful predictor of AF ablation. Of note, the AP LA diameter was not an accurate depiction of the true LA size with TTE [15]. The authors reported that the AP LA diameter determined using TTE was not a predictor of AF ablation outcomes [8]. In this study, we found a moderate correlation between the LA diameter determined using TTE and the LA volume determined using MSCT (*r* = 0.594, *p* < 0.001; Figure 4). MSCT provides a more rigorous evaluation of the bona fide LA size, having a higher accurate value for long-term outcomes after RFCA for AF. Notably, several studies using LA volume instead of the AP LA diameter found a strong correlation between LA size and AF recurrence after RFCA [8,11,17].

A shorter AF duration is associated with easier termination of the episode. The type of AF is one of the most consistent predictors of long-term outcomes after RFCA and is also the only prognostic factor affecting the level of recommendation of RFCA in current guidelines, which means PeAF like larger LA size has been shown to be associated with a poorer long-term outcome after ablation when compared with PAF [17,29]. Our data unequivocally demonstrated an elevated incidence and odds ratio of AF recurrence among patients with PeAF. An increased LA volume, commonly observed in PeAF, is generally representative of advanced structural remodeling [30]. This complexity is associated with more challenging conditions for catheter ablation and correlates with an increased rate of atrial fibrillation recurrence. In our study, the median LA volume in patients with PAF was remarkably smaller than in those with PeAF. However, Francisco et al. reported that there was a sizable overlap in the median LA volumes between PAF and PeAF patients undergoing RFCA and that the LA volume was the most valuable parameter of recurrence after RFCA, overshadowing AF type as a prognostic factor [12].

In this study, we also found that a larger LA volume based on MSCT images was associated with AF recurrence after RFCA. Especially, LA volume ≥ 153.2 mL could strongly predict AF recurrence after RFCA, suggesting that substantial LA remodeling is a strong predictor of recurrent AF. Abecasis et al., who reported a significantly increased probability of AF recurrence in patients with a computed tomography-derived LA volume ≥ 100 mL, also found that the optimal cutoff value for LA volume was 145 mL [8]. The reported LA volume cutoff values to predict AF recurrence vary across different studies [11,12,13]. D’Ambrosio et al. described a 57 mL/m^2^ cutoff value for LA volume as a predictor of low-voltage areas in the left atrium [31]. We predict that a narrow range of LA volume might have better utility than one fixed value as a cutoff, although the cutoff value for LA volume determined in the current study should be confirmed in future prospective studies.

Multiple trials showed that successful ablation might result in improved LV function, clinical heart failure status, quality of life, and so on. However, the efficacy of RFCA in heart failure patients with AF according to LVEF is still controversial until now. Our study reported that the AF recurrence rate was lower in patients with higher LVEF within the normal LVEF range. Recent studies including meta-analysis trials have confirmed that LVEF did not have a significant relationship with AF recurrence after RFCA and patients with and without LVSD had similar risk for recurrence rate after catheter ablation [32,33].

Pinto et al. suggested that LAA volume measured using computed tomography is also an important predictor of AF recurrence after the first RFCA and that LAA volumes > 8.825 mL exhibited a good correlation in predicting AF recurrence [17]. In contrast to that study, LAA volume was not a predictor of AF recurrence after RFCA in the current study. This discrepancy might be related to the more complex morphology of LAA compared with that of the left atrium, which might hinder accurate volume measurements, or to the relatively minor role of LAA in triggering or maintaining AF compared to the PV.

## 5. Limitations

The present work has several limitations. First, its results are based on a single-center study with retrospective data analysis. Although all patients were closely followed with regular clinic appointments and ECG and Holter monitoring, asymptomatic AF episodes might have been missed. The sample size was modest, and a limited number of patients with non-paroxysmal AF precluded the performance of a high statistical power comparison between the type of AF and outcomes. Additional limitations are related to the routine use of MSCT for evaluating cardiac anatomy and LA volume. Gating is a prevailing limiting factor in patients with PeAF who experience irregular heart rates. As iodinated contrast is used during MSCT, patients with decreased kidney function should be hydrated to prevent contrast nephropathy. The radiation dose remains a concern, especially in young female patients [34]. In patients with concerns regarding the usefulness of MSCT, magnetic resonance imaging provides information on PV anatomy and precise measurement of LA volume avoiding radiation exposure [35]. Finally, we are afraid that we did not achieve the long-term F/U date, as more than half of patients were not followed up.

## 6. Conclusions

Evaluation with cardiac MSCT before RFCA is a tool for the objective and observer-independent determination of anatomical remodeling and LA volume. While we found a significant relationship between LA volume one-year outcomes after AF ablation, the association was not linear. LA volume ≥ 153.2 mL had good accuracy in predicting AF recurrence after RFCA, whereas there was no relationship between LAA volume and outcomes following RFCA for AF. PeAF has also been shown to be associated with a poor prognostic factor after ablation. These findings should be confirmed via further studies including patients with different AF types to determine the optimal cutoff value for LA volume using MSCT.

## Figures and Tables

**Table 1:** Baseline characteristics of study patients based on recurrence after ablation for atrial fibrillation.

	All Patients (*n* = 152)	No Recurrence (*n* = 118)	Recurrence (*n* = 34)	*p*
Age (years)	55.8 ± 9.6	55.9 ± 9.5	55.1 ± 10.2	0.624
Male, *n* (%)	110 (72.4)	85 (72.0)	25 (73.5)	1.000
BMI (kg/m^2^)	25.4 ± 3.3	25.2 ± 2.9	25.7 ± 4.3	0.428
PAF, *n* (%)	99 (65.1)	87 (73.7)	12 (35.3)	<0.0001
Hypertension, *n* (%)	53 (34.9)	41 (34.7)	12 (35.3)	1.000
Diabetes, *n* (%)	11 (7.2)	9 (7.6)	2 (5.9)	1.000
Stroke, *n* (%)	15 (9.2)	8 (6.8)	6 (17.6)	0.086
LA volume (mL)	142.5 ± 33.7	139.2 ± 34.1	153.8 ± 29.9	0.025
LAA volume (mL)	15.1 ± 6.5	14.7 ± 6.5	16.2 ± 6.3	0.235
LVEF (%)	58.9 ± 6.7	59.9 ± 5.1	55.5 ± 6.2	0.001

BMI, body mass index; LA, left atrial; LAA, left atrial appendage; LVEF, left ventricular ejection fracture; PAF, paroxysmal atrial fibrillation.

**Table 2:** Predictors of AF recurrence after catheter ablation based on univariable Cox regression analysis.

	Adjusted Hazard Ratio	95% Confidence Interval	*p* Value
Age	0.992	(0.957, 1.029)	0.674
Sex	1.135	(0.529, 2.434)	0.745
BMI	1.04	(0.935, 1.158)	0.469
Hypertension	1.029	(0.509, 2.08)	0.936
Diabetes	0.772	(0.185, 3.225)	0.723
Stroke	2.188	(0.905, 5.291)	0.082
PeAF	3.944	(1.95, 7.975)	0.001
LA volume	1.009	(1, 1.018)	0.063
LAA volume	1.021	(0.976, 1.068)	0.359
LAA/LA ratio	0.327	(0, 560.421)	0.769
LA volume ≥ 153 mL	2.771	(1.385, 5.541)	0.004
LVEF	0.939	(0.906, 0.974)	0.001
Number of AAD	1.223	(0.927, 1.614)	0.155

AF, atrial fibrillation; BMI, body mass index; LA, left atrium; LAA, left atrial appendage; LVEF, left ventricular ejection fracture; PeAF, persistent atrial fibrillation.

**Table 3:** Predictors of AF recurrence after adjustment for PeAF, LA volume ≥ 153 mL, and LVEF based on multivariable Cox regression analysis.

	Adjusted	95%	*p*
	Hazard Ratio	Confidence Interval	
PeAF	2.759	(1.26, 6.041)	0.011
LVEF	0.964	(0.927, 1.002)	0.065
LA volume ≥ 153 mL	1.616	(0.752, 3.476)	0.219

AF, atrial fibrillation; PeAF, persistent atrial fibrillation; LVEF, left ventricular ejection fracture; LA, left atrium.

## Data Availability

No new data were created or reported in this study.
